# Adverse Reactions after Administration of Antivenom in Korea

**DOI:** 10.3390/toxins12080507

**Published:** 2020-08-06

**Authors:** Jin Seok Shim, Hyunggoo Kang, Yongil Cho, Hyungoo Shin, Heekyung Lee

**Affiliations:** 1 Department of Emergency Medicine, College of Medicine, Hanyang University Hospital, Hanyang University, 04763 Seoul, Korea; paulpinic@naver.com (J.S.S.); Massdt@naver.com (H.L.); 2 Department of Emergency Medicine, College of Medicine, Hanyang University Guri Hospital, Hanyang University, 11923 Guri, Korea; seodtst@gmail.com

**Keywords:** snake venom, antivenom, adverse reaction, snakebite

## Abstract

Kovax^®^ antivenom is the main treatment for toxins produced by the *Gloydius* species. However, research on adverse reactions after Kovax^®^ antivenom administration is scarce. We aimed to identify the incidence and characteristics of adverse reactions after Kovax^®^ antivenom administration. We conducted a retrospective review of the medical records of snakebite patients in Korea between January 2008 and September 2019. We identified the frequency, characteristics, and treatments of adverse reactions to Kovax^®^ antivenom. There were 150 patients with snakebites, of whom 121 (80.7%) patients received Kovax^®^ antivenom. Adverse reactions occurred in five patients (4.1%). Acute adverse reactions within 24 h of antivenom administration occurred in two patients (1.7%). The symptoms of patients with acute adverse reactions were nausea, diaphoresis, dizziness, and hypotension. Delayed adverse reactions that occurred 24 h after antivenom administration were reported in three patients (2.5%). One patient had a skin rash after 10 days, and two patients had fever 37 and 48 h after antivenom use. In conclusion, most patients were managed safely after Kovax^®^ antivenom, and the incidence of adverse reactions was low. Severe adverse reactions occurred in a small percentage of patients, and there were no deaths.

## 1. Introduction

There are approximately 3000 species of snakes worldwide, of which 600 are venomous [1]. According to data released by the WHO, 421,000 to 2.5 million patients are bitten by venomous snakes, of whom 20,000 to 100,000 die each year [2]. Of the 600 species of venomous snakes, four are found in Korea: *Gloydius brevicaudus*, *Gloydius ussuriensis*, and *Gloydius intermedius*, belonging to the Viperidae family, and *Rhabdophis tigrinus*, belonging to the Colubridae family [3]. In the Gloydius species, many components of venom that cause coagulopathy have been identified. In the venom of *Gloydius brevicaudus*, the following components have been identified: salmosin, a platelet glycoprotein IIb-IIIa [4]; brevinase, a fibrinolytic enzyme [5]; halyxin, which inhibits coagulation [6]; halysase, which inhibits platelet aggregation [7]; and salmorin, which inhibits fibrinogen clotting [8]. A thrombin-like enzyme called calobin has been found in *Gloydius ussuriensis* [9,10]. Saxatilin, extracted from *Gloydius intermedius*, strongly inhibits the activation and aggregation of platelets and has thrombolytic effects [11]. According to big data from the Health Insurance Review & Assessment Service in Korea, on average 2315 to 4143 patients are affected annually by bites from venomous snakes in South Korea [12]. Although there are slight differences depending on the severity of snakebite, region and hospital, antivenom is used in 70–80% of snakebite patients [13,14].

The use of antivenom is the most important treatment for snakebites. The WHO recommends using antivenom if local swelling worsens or hemostatic abnormalities or organ injury develop. Antivenoms of various qualities and prices are used worldwide [2,15]. However, the use of antivenom can cause various adverse reactions, so it should be administered carefully. Severe adverse reactions can develop within an hour after the administration of antivenom, and the patient should be observed; vital signs should be monitored to detect adverse reactions [3,16]. Antivenom safety depends on manufacturing factors, such as immunoglobulin composition, purification of immunoglobulin fragments, protein concentration, and presence of preservatives [15]. Initial antivenom used whole immunoglobulin G (IgG) containing both fragment crystallizable (Fc) and fragment antigen-binding (Fab) regions and showed many adverse reactions [17]. Antivenoms consisting of Fab or F(ab’)2 with Fc fragments removed from IgG were developed to reduce adverse reactions. However, it was found that the purity and the protein content of antivenom rather than Fc-mediated complement activation were related to adverse reactions. In fact, well-purified whole IgG antivenom shows no difference in potency or safety from F(ab’)2 antivenom [18]. The incidence rate of adverse reactions to antivenom varies based on the technology used to purify it, the region and the type of antivenom. In Japan, where high-quality antivenom is used, the incidence of adverse antivenom reactions is reported to be 2.4–8% [19]. In Europe, the adverse reaction rate is approximately 1.5%, suggesting that modern intravenous antivenoms are of good quality [20]. In many regions, there are no minimum specifications for the potency, efficacy, dose or safety of antivenom products and quality-assured products are not available due to poor control and regulation of preparations [21]. Especially in Africa and South-east Asia, weak regulatory frameworks create a situation where ineffective products enter without evaluation and strictly regulated competitors give up production [22,23,24]. In Southeast Asia, a high incidence of adverse reactions is reported (30–80%) [25]. In addition, a study published in Australia in 2008 reported that approximately 25% of patients experience adverse events, of which 5% are serious [26].

An injection of Kovax^®^ freeze-dried *Gloydius brevicaudus* antivenom (KOREAVACCINE Co., Ltd., Korea; 6000 units/vial) is the primary treatment for snakebites by *Gloydius* species in Korea. *Gloydius* is a genus of venomous pit vipers in Asia, formerly called *Agkistrodon*. The newly named genus *Gloydius* is very similar to *Agkistrodon* in North America and has 22 recognized species. Kovax^®^ antivenom is the only antivenom used in Korea [27], and it is necessary to know about its safety and the frequency of adverse reactions to Kovax^®^ antivenom. However, research on the epidemiology of adverse reactions is scarce. Therefore, we aimed to identify the incidence and characteristics of adverse reactions after antivenom administration in Korea. We investigated acute reactions, including pyrogenic and anaphylactic reactions, as well as delayed reactions, referred to as serum sickness.

## 2. Results

### 2.1. Characteristics of the Study Population

The baseline characteristics of the patients are summarized in Table 1. A total of 150 snakebite patients visited two emergency medical centers from January 2008 to September 2019. The mean age of the patients was 52.1 ± 17.6 years, and male patients comprised 65.3% (*n* = 98) of the study population. Among them, a total of 121 patients received antivenom. Twelve patients with grade I bites did not meet the indications of the WHO guidelines but received antivenom as determined by their physicians. More than half of the patients had grade III (34.7%; *n* = 52) or grade II (28%; *n* = 42) bites. A total of 23 (15.3%) patients had neurological symptoms, including 17 patients with diplopia and six patients with visual disturbance. Venom-induced consumptive coagulopathy (VICC) occurred in 13.3% (*n* = 20) of the patients, and thrombocytopenia occurred in 14.0% (*n* = 21). Bleeding complications occurred in 3.3% (*n* = 5) of the patients: one patient with hemoperitoneum, two patients with gastrointestinal bleeding, one patient with epistaxis and gingival bleeding, and one patient with hemoptysis. The mean hospital length of stay was 3.1 days. None of the 150 patients died in the hospital.

### 2.2. Adverse Reactions after Antivenom Administration

The dose of antivenom and frequency of adverse reactions are described in Table 2. The mean initial and total doses of antivenom were 6124 units and 10,240 units, respectively. In most cases, no additional dose was administered after the initial 6000 unit dose. Patients with an advanced-grade snakebite or complications such as VICC and bleeding received additional doses of antivenom. A total of 54,000 units of antivenom was the highest dose.

Adverse reactions occurred in five patients (4.1%). Acute adverse reactions occurred in two patients (1.7%), and delayed reactions occurred in three patients (2.5%). The symptoms in patients with acute adverse reactions were nausea, diaphoresis, dizziness, and hypotension. The symptoms in patients with delayed adverse reactions were skin rash and fever. The clinical characteristics of patients with acute and delayed adverse reactions are summarized in Table 3 and Table 4.

### 2.3. Clinical Details of Patients with Acute Adverse Reactions: Case 1

A 62-year-old man visited an emergency medical center after a snakebite (Table 3, patient 1). His right hand was bitten by a snake of unknown species, and pain and swelling progressed beyond the wrist (grade III). His initial vital signs were as follows: blood pressure, 127/76 mmHg; pulse rate, 77 beats per minute; respiration rate, 18 breaths per minute; and body temperature, 36.6 °C. He was alert, and he had no neurological symptoms. The laboratory blood tests did not show any specific findings. He received Kovax^®^ antivenom at a rate of 50 units/min. After 80 min, the patient complained of nausea, dizziness and diaphoresis. His heart rate was 60 beats per minute, his blood pressure had dropped to 88/55 mmHg, and the administration of the antivenom was immediately discontinued. After the bolus administration of 500 mL of crystalloid, his heart rate and blood pressure recovered to 67 beats per minute and 122/79 mmHg, respectively. He did not receive antihistamines, corticosteroids, or adrenaline, and his vital signs remained stable without other symptoms. Hospitalization of the patient was recommended, but he refused and was discharged against medical advice.

### 2.4. Clinical Details of Patients with Acute Adverse Reactions: Case 2

A 65-year-old man working in a cemetery was bitten by a snake and visited the emergency room one hour after the snakebite (Table 3, patient 2). A photograph was taken of the snake immediately after it bit him, and it was confirmed that it was *Gloydius intermedius*. The site of the snake bite was the fourth finger on the right hand, and edema progressed to the forearm. His initial vital signs were as follows; blood pressure, 149/91 mmHg; pulse rate, 68 beats per minute; respiration rate, 18 breaths per minute; and body temperature 37.7 °C. He was alert, and he had no neurological symptoms. On the initial laboratory blood test, his platelet level had fallen to 98,000 (count/mm^3^), and his D-dimer level had risen to 1.13 mg/L. He received Kovax^®^ antivenom at a rate of 40 units/min. Thirty minutes after the administration of antivenom was started, he complained of nausea, dizziness, and diaphoresis. His blood pressure had dropped to 80/60 mmHg, and the administration of antivenom was stopped. To treat the acute reaction, 4 mg of chlorpheniramine and 62.5 mg of methylprednisolone were administered intravenously, and 0.3 mg of adrenaline was injected intramuscularly. After the injection, his symptoms improved, his blood pressure was 111/67 mmHg, and his heart rate was 73 beats per minute. He was transferred to the other emergency department included in our study. When he arrived there 8 h after receiving the snakebite, the swelling had advanced beyond the right upper arm, his platelet count was 74,000 (platelets/mm^3^), his D-dimer level was 8.81 mg/L, and his prothrombin time (PT) and activated partial thromboplastin time (aPTT) were not measurable. He was hospitalized in the emergency ward, and by hospital day (HD) 4, the swelling and bruising had advanced to the thigh and scrotum. Since an adverse reaction had occurred after the initial use of antivenom, no additional antivenom was used until this time. He complained of hemoptysis on HD 8. He was transfused with cryoprecipitate, packed red blood cells, platelets, and purified fibrinogen, but the VICC and thrombocytopenia did not improve. It was decided to administer antivenom while monitoring the patient in the ICU. Eight milligrams of chlorpheniramine and 100 mg of hydrocortisone were administered as a pretreatment 30 min before the antivenom administration. Antivenom was administered as 6000 units in 500 mL of normal saline at a rate of 8 units/min on HDs 8 and 9. No adverse reactions occurred, his clinical condition improved, and he was discharged on HD 18.

### 2.5. Clinical Details of Patients with Delayed Adverse Reactions

Among the three patients with delayed adverse reactions, one had a skin rash, and two had a fever. For the patient with a skin rash (Table 4, patient 1), the first 6000 units of antivenom were given on the day of the snakebite, and 6000 units of antivenom were administered on HDs 4 and 5. Ten days after the first antivenom administration, erythematous wheals were observed on both arms, legs, and buttocks, and the patient’s vital signs were stable. To treat the rash, antihistamines and corticosteroids were administered, and topical steroids were applied. In the other two patients with a fever, one patient received 6000 units of antivenom on the day of the snakebite and the next day, and she developed a 38.0 °C fever 48 h after the first antivenom administration (Table 4, patient 2). Another patient had a 38.1 °C fever 37 h after the administration of 6000 units of antivenom on the day of the snakebite (Table 4, patient 3). Antipyretics were used in both patients to treat the fever.

## 3. Discussion

In this study, antivenom was used in 81% (121/150) of the patients, and adverse reactions occurred in 4.1% after the administration of Kovax^®^ antivenom. Acute and delayed adverse reactions occurred in 1.7% and 2.5% of the patients, respectively. There were no deaths after Kovax^®^ antivenom administration. There have been several Korean papers containing data on the adverse events of patients using Kovax^®^ antivenom. You et al. reported that 16 out of 62 snakebite patients used antivenom and three had delayed adverse events (two skin rash, one serum sickness) [28]. In studies by Jin et al. [29] and Jun et al. [30], there were 87 and 94 snakebite patients with Grade 2 or higher who were presumed to have been administered antivenom. Of the 181 patients in these two studies, one patient with serum sickness was reported. When these three studies were combined, 4 out of 197 patients who received antivenom had delayed adverse events (two skin rash, two serum sickness). This is a number similar to that in our study. However, these studies were not focused on the use and adverse events of antivenom, and the numbers might not be accurate. According to studies conducted in other countries, adverse reactions can occur in 2% to 88% of patients, depending on the year, country, environment, and type of antivenom used [31,32]. According to Mahasandana et al., 20.8% of 178 patients who received green pit viper antivenom in Thailand experienced adverse reactions [33]. Thiansookon et al. reported that 2.3% and 12.5% of patients who received lyophilized equine F (ab’)2, species-specific antibodies after being bitten by green pit vipers and cobras experienced adverse reactions [34]. Mong et al. reported that adverse reactions caused by green pit viper antivenom and *A. halys* antivenom occurred in 4.7% and 1.4% of patients, respectively, in Hong Kong [35]. According to Kleinschmidt et al., the rate of side effects of Crotalidae Polyvalent Immune Fab in the North American snakebite registry was 2.7%. Ryan et al. reported acute adverse reactions in 23% of patients and serum sickness in 29% in the Australian snakebite project [36]. The incidence of adverse reactions in our study tended to be lower than those reported in other studies.

Acute reactions are divided into pyrogenic reactions and anaphylactic reactions. Pyrogenic reactions usually occur within hours of the injection of antivenom and are caused by pyrogenic substances contaminating the antivenom [2,16,37]. In our study, there were two patients with anaphylactic reactions, and both showed hypotension, dizziness, and diaphoresis. Anaphylactic reactions are divided into immunoglobulin E (IgE)-mediated and non-IgE-mediated reactions. IgE-mediated reactions occur when IgE antibodies bind to mast cells and basophils. During antivenom administration, IgE antibodies that interact with Fc3 receptors present in mast cells and basophils recognize the antivenom proteins and induce cell degranulation [16,37]. As cell degranulation is induced, chemicals such as leukotriene and prostaglandin are secreted [37]. These chemical substances induce vasodilatation, increase permeability within tens of minutes, contract the smooth muscles, and increase the function of the glands. Non-IgE-mediated reactions account for most of the acute reactions caused by antivenom and are still incompletely understood. Non-IgE-mediated reactions are described as occurring via two mechanisms: antivenom anticomplementary activity (ACA) and the presence of heterophilic antibodies. Because these reactions are not IgE-mediated, an intradermal hypersensitivity test is not recommended, as it is useless for predicting occurrence [2,3,37]. It is difficult to determine whether the acute reactions in our study were IgE-mediated or non-IgE-mediated reactions. In general, Kovax^®^ antivenom uses whole IgG containing Fc fragments, so non-IgE-mediated immune responses are likely to occur [3].

Delayed reactions caused by antivenom are also referred to as serum sickness. Clinically, fever, itchy urticaria, arthralgia, etc., occur as symptoms and can occasionally cause proteinuria with immune complex nephritis and encephalopathy [2,16]. In delayed reactions, the human immune system recognizes the antivenom as heterologous proteins and produces IgG-based antibodies [37]. The concentration of antibodies in the serum against heterologous immunoglobulins increases by 2- to 100-fold or more compared to baseline values after antivenom administration [37,38]. The formation of soluble antigen–antibody complexes is responsible for the reaction, which generally manifests clinically between five and 20 days after antivenom administration [2,16,36,37]. In our study, there was one patient with a skin rash on the 10th day after antivenom administration. There were no cases of acute pyrogenic reactions after antivenom administration, and two patients developed fever on the second day after antivenom administration. It is not known exactly why fewer pyrogenic reactions are reported, but it can be assumed that pyrogens are well removed by the manufacturing process of Kovax^®^ antivenom [39].

For the treatment of adverse reactions, crystalloid hydration and drug treatment were provided based on the World Allergy Organization Anaphylaxis Guidelines [16]. In the first case in our study, the patient recovered from the adverse reaction only when the administration of antivenom was discontinued and a bolus of crystalloid was administered. In the second case, the patient recovered from the adverse reaction after receiving antihistamines, corticosteroids, and adrenaline. This patient received antivenom again for the treatment of sustained complications. Antihistamines and corticosteroids were administered as a pretreatment, the administration rate was slowed, and no adverse reaction occurred. However, there have been reports that the use of adrenaline can reduce the occurrence of adverse reactions, so it is worth considering adrenaline as a pretreatment in places with high rates of adverse reactions [16,25,40]. According to previous studies, the use of antihistamines and corticosteroids has been reported to have no effect as a pretreatment [25,35,41,42,43]. In a randomized controlled trial conducted in Sri Lanka, the rate of administration did not affect the occurrence of adverse reactions [44]. In our study, adrenaline was not used as a pretreatment, and no adverse reactions occurred after slowing the rate of antivenom administration in the second case. Because of the relatively small sample size, it is difficult to conclude that adjusting the administration rate is effective for reducing the occurrence of adverse reactions to antivenom, but it is worth considering in patients who are absolutely in need of antivenom administration. There are no local guidelines regarding the use of antivenom for snakebites in Korea. Further studies of the indications and appropriate dosages of antivenom according to severity are needed, and guidelines should be established accordingly.

There have been reports that the need for skin tests before the administration of antivenom is controversial but that skin tests should be performed until clear instructions are given [45]. However, Chuang et al. reported that the skin test has high specificity but extremely low sensitivity, failing to help predict adverse reactions to antivenom [46]. There are no research data on this topic in Korea, but it is recommended not to perform skin tests or desensitization; instead, treatment is recommended if adverse reactions to antivenom occur [3]. Considering the low incidence of adverse reactions in our study and the above reports, there is no clear evidence supporting the routine use of skin tests. However, it is necessary to closely monitor the patient’s symptoms and vital signs when administering antivenom [2,3,37].

Our study has some limitations. First, it was conducted with data collected from two hospitals in South Korea, and there are limitations regarding generalization of the results. This study included only a very small fraction of patients treated with Kovax^®^ antivenom. However, we could assume that adverse reactions to antivenom used in Korea are rare. Further study of more patients is needed to provide a better comparative framework. Second, since this was a retrospective study based on medical records, all information needed may not have been recorded. Third, delayed adverse reactions usually appear 5–14 days after antivenom administration; however, most patients who received antivenom were discharged after an average hospitalization period of approximately 3.5 days. Therefore, there is a difference between the incidence of delayed adverse reactions confirmed in our study and the actual incidence of delayed adverse reactions.

## 4. Conclusions

In our study, approximately 80% of patients were administered antivenom, and the incidence of adverse reactions to antivenom was found to be low (4.1%). Reducing the rate of antivenom administration and pretreatment may help reduce the occurrence of adverse reactions, but further investigation is needed for confirmation.

## 5. Materials and Methods

### 5.1. Study Design and Setting

We conducted a retrospective review of the medical records of snakebite patients who visited emergency medical centers at two university hospitals in Korea between January 2008 and September 2019. Currently, Kovax^®^ antivenom is produced with whole IgG antibodies and is obtained from horses injected with snake venom from *Gloydius brevicaudus* [3]. Kovax^®^ antivenom is the main treatment for neutralization of the venom of the *Gloydius* species present in Korea, *Gloydius brevicaudus*, *Gloydius ussuriensis*, and *Gloydius intermedius*, because there are common antigens among snake venoms originating from the genus *Gloydius*. Kovax^®^ antivenom can be administered intravenously after dilution with saline, or it can be injected intramuscularly. The initial dose of Kovax^®^ antivenom is 6000 units, and 3000–6000 units can be injected after two to three hours when symptoms are not relieved. One unit of antivenom is an anti-lethal or anti-hemorrhagic titer that neutralizes one test dose of venom. The lethal and hemorrhagic titer of the national reference standard of *Gloydius* snake venom is 90.13 μg and 10.80 μg per 1 test dose, respectively [47]. One vial of Kovax^®^ antivenom contains more than 6000 anti-lethal and anti-hemorrhagic units, respectively. Data were collected from patients prescribed Kovax^®^ antivenom and those who had a diagnostic code for the toxic effects of snake venom (International Classification of Diseases-10 code: T630). The institutional review boards of Hanyang University Hospital and Hanyang University Guri Hospital in Korea approved the study on 29 October 2019 and 14 January 2020, respectively. (IRB Nos. HYUH 2019-10-034; GURI 2019-12-040).

### 5.2. Data Collection

We collected data regarding demographics, location of injury, symptoms, and grade classification of the study population. We classified the injury severity into five groups based on the Mamushi grade classification [13,48,49]: I, redness and swelling around the area of the bite; II, redness and swelling of the wrist or foot joint; III, redness and swelling of the elbow or knee joint; IV, redness and swelling of the whole extremity; and V, redness and swelling of parts beyond the extremity or systemic symptoms.

The outcomes of interest in this study were the frequency and symptoms of adverse reactions to antivenom administration. WHO guidelines recommend antivenom for the following indications [2]: (1) systemic envenomation, such as neurotoxic signs, hemostatic abnormalities, cardiovascular abnormalities, myoglobinuria, hemoglobinuria, and acute kidney injury; (2) local swelling involving more than half of the bitten limb; and (3) the rapid expansion of swelling. We confirmed the rate and dose of antivenom administration. Antivenom adverse reactions were investigated. We confirmed that the following adverse reactions occurred after antivenom administration: nausea, vomiting, diarrhea, dyspnea, hypotension, tachycardia, dizziness, diaphoresis, skin rash, and fever [2,35]. In our study, acute adverse reactions were defined as those occurring within 24 h after the administration of antivenom, and delayed adverse reactions were defined as reactions occurring more than 24 h after the administration of antivenom [2,16,36,37].

Coagulopathy is caused by the inhibition of coagulation factors and the consumption of coagulation factors secondary to the promotion of coagulation. This characteristic is called VICC, with pathway involvement similar to that of disseminated intravascular coagulopathy (DIC) [50,51]. The definition of and diagnostic criteria for VICC were obtained from a previous study [52]. Thrombocytopenia was defined as a platelet count below the lower limit of normal (150,000 platlets/mm^3^) [13,53].

### 5.3. Data Analysis

The general characteristics of the patients included age, sex, location of snakebite, grade of snakebite, symptoms and complications after snakebite, and length of hospital stay. In the antivenom group, we collected data on the dose of antivenom, the type and incidence of adverse reactions, and the treatment of adverse reactions. Categorical variables were reported as frequencies and percentages. The normality of the distribution of continuous variables was tested with the Shapiro-Wilk test. Depending on the results, continuous variables are presented as the means and standard deviations or medians and interquartile ranges. All data were collected in Microsoft Excel (Microsoft Corp., Redmond, WA, USA) and analyzed with R version 3.4.0 (www.R-project.org).

## Figures and Tables

**Table 1:** Characteristics of the study population.

	Total Snake Bite Patients (*n* = 150)	Antivenom Use (*n* = 121)	No Antivenom Use (*n* = 29)
*Age*, *mean (SD), years*	52.1 (17.6)	52.4 (18.0)	50.5 (15.7)
*Sex, no. (%)*			
Male	98 (65.3)	79 (65.3)	19 (65.5)
Female	52 (34.7)	42 (34.7)	10 (34.5)
*Location of injury, no. (%)*			
Upper extremity	87 (58.0)	72 (59.5)	15 (51.7)
Lower extremity	63 (42.0)	49 (40.5)	14 (48.3)
*Grade, no. (%)*			
I	35 (23.3)	12 (10.0)	23 (79.3)
II	42 (28.0)	38 (31.4)	4 (13.8)
III	52 (34.7)	50 (41.3)	2 (6.9)
IV	16 (10.7)	16 (13.2)	0 (0.0)
V	5 (3.3)	5 (4.1)	0 (0.0)
*Neurological symptom, no. (%)*	23 (15.3)	23 (19.0)	0 (0.0)
*Cardiovascular symptom, no. (%)*	2 (1.3)	2 (1.7)	0 (0.0)
*Hematological complication, no. (%)*			
Venom-induced consumption coagulopathy	20 (13.3)	20 (16.5)	0 (0.0)
Thrombocytopenia	21 (14.0)	20 (16.5)	1 (3.4)
Bleeding	5 (3.3)	5 (4.1)	0 (0.0)
Transfusion	17 (11.3)	17 (14.0)	0 (0.0)
Fibrinogen concentrate	4 (2.7)	4 (3.3)	0 (0.0)
*Hospital length of stay*, *mean (SD), days*	3.1 (4.1)	3.5 (4.2)	1.2 (3.0)
*Survival to discharge, no. (%)*	150 (100)	121 (100)	29 (100)

SD = standard deviation.

**Table 2:** Use of antivenom and frequency of adverse reactions.

	Antivenom Use (*n* = 121)
*Antivenom use*	
Initial dose of antivenom, *mean (SD),* units	6124 (1469.5)
Total dose of antivenom, *mean (SD),* units	10,240 (7664.9)
*Acute adverse reaction, no. (%)*	2 (1.7)
Hypotension	2 (1.7)
Diaphoresis	2 (1.7)
Nausea	2 (1.7)
Dizziness	2 (1.7)
Bradycardia, tachycardia	0 (0)
Dyspnea	0 (0)
Skin rash, angioedema	0 (0)
Fever	0 (0)
*Delayed adverse reaction, no. (%)*	3 (2.5)
Skin rash	1 (0.8)
Fever	2 (1.7)

SD = standard deviation.

**Table 3:** Clinical details of patients with acute adverse reactions associated with the administration of antivenom.

Characteristics	Patient No.	
	1	2
*Age (years)/Sex*	62/Male	65/Male
*Comorbidities*	None	None
*Snake species*	Unknown	*Gloydius intermedius*
*Bite site*	Hand	Hand
*Grade classification*	III	V
*Symptoms*	Hand pain, dizziness	Hand pain, swelling
*Initial vital signs*		
SBP/DBP (mmHg)	127/76	149/91
HR (beats/min)	77	68
RR (breaths/min)	18	18
BT (°C)	36.6	37.7
Blood oxygen saturation (%)	100	100
Mental status	Alert	Alert
*Antivenom administration*		
Location	ED	ED
Pretreatment	No	No
Infusion rate (units/min)	50	40
Dose (units)	4000	1200
*Adverse reactions after antivenom*		
Onset (min)	80	30
Symptoms	Nausea, dizziness, diaphoresis, hypotension	Nausea, dizziness, diaphoresis, hypotension
*Vital signs after adverse reactions*		
SBP/DBP (mmHg)	88/55	80/60
HR (beats/min)	60	65
RR (breaths/min)	18	18
BT (°C)	Not checked	Not checked
Blood oxygen saturation (%)	100	100
Mental status	Alert	Alert
Treatment for adverse reactions	Supportive care	Antihistamines, corticosteroids, adrenaline
*Additional antivenom*	No	Yes
Dose (units)		12,000
Location		ICU

SBP = systolic blood pressure; DBP = diastolic blood pressure; HR = heart rate; RR = respiratory rate; BT = body temperature; ED = emergency department, ICU = intensive care unit.

**Table 4:** Clinical details of patients with delayed adverse reactions associated with the administration of antivenom.

Characteristics	Patient No.		
	1	2	3
*Age (years)/Sex*	41/Female	89/Female	51/Male
*Comorbidities*	None	HTN, DM	DM
*Snake species*	*Gloydius brevicaudus*	Unknown	Unknown
*Bite site*	Foot	Foot	Foot
*Grade classification*	III	III	III
*Symptoms*	Foot pain	Foot pain	Foot pain
*Antivenom administration*			
Location	ED	ED	ED
Pretreatment	None	None	None
Infusion rate (units/min)	20	50	16
Dose (units)	6000	6000	6000
*Adverse reactions after antivenom*			
Onset (hours)	240	48	37
Symptoms	Skin rash	Fever	Fever
*Vital signs after adverse reactions*			
SBP/DBP (mmHg)	100/60	110/60	130/90
HR (beats/min)	70	80	80
RR (breaths/min)	18	20	20
BT (°C)	37.2	38.0	38.1
Blood oxygen saturation (%)	100	100	100
Mental status	Alert	Alert	Alert
Treatment for adverse reactions	Antihistamines, corticosteroids, topical steroids	Antipyretics	Antipyretics
*Additional antivenom*	Yes	Yes	No
Dose (units)	12,000	6000	
Location	Ward	Ward	

HTN = hypertension; DM = diabetes mellitus; ED = emergency department; SBP = systolic blood pressure; DBP = diastolic blood pressure; HR = heart rate; RR = respiratory rate; BT = body temperature.
